# 5mC modification patterns provide novel direction for early acute myocardial infarction detection and personalized therapy

**DOI:** 10.3389/fcvm.2022.1053697

**Published:** 2022-12-23

**Authors:** Yiqun Guo, Hua Jiang, Jinlong Wang, Ping Li, Xiaoquan Zeng, Tao Zhang, Jianyi Feng, Ruqiong Nie, Yulong Liu, Xiaobian Dong, Qingsong Hu

**Affiliations:** ^1^Department of Interventional Radiology and Vascular, Guangzhou Women and Children’s Medical Center, The Affiliated Hospital of Guangzhou Medical University, Guangzhou, Guangdong, China; ^2^Department of Cardiology, The First Affiliated Hospital of Jinan University, Guangzhou, Guangdong, China; ^3^Department of Cardiology, Xinfeng County People’s Hospital, Shaoguan, Guangdong, China; ^4^Department of Cardiology, Sun Yat-sen Memorial Hospital, Sun Yat-sen University, Guangzhou, Guangdong, China; ^5^Department of Intervention and Vascular Surgery, The First Affiliated Hospital of Jinan University, Guangzhou, Guangdong, China

**Keywords:** acute myocardial infarction, coronary heart disease, epigenetics, bioinformatics, 5mC

## Abstract

**Background:**

Most deaths from coronary artery disease (CAD) are due to acute myocardial infarction (AMI). There is an urgent need for early AMI detection, particularly in patients with stable CAD. 5-methylcytosine (5mC) regulatory genes have been demonstrated to involve in the progression and prognosis of cardiovascular diseases, while little research examined 5mC regulators in CAD to AMI progression.

**Method:**

Two datasets (GSE59867 and GSE62646) were downloaded from Gene Expression Omnibus (GEO) database, and 21 m5C regulators were extracted from previous literature. Dysregulated 5mC regulators were screened out by “limma.” The least absolute shrinkage and selection operator (LASSO) and support vector machine recursive feature elimination (SVM-RFE) algorithm were employed to identify hub 5mC regulators in CAD to AMI progression, and 43 clinical samples (Quantitative real-time PCR) were performed for expression validation. Then a logistic model was built to construct 5mC regulator signatures, and a series of bioinformatics algorithms were performed for model validation. Besides, 5mC-associated molecular clusters were studied *via* unsupervised clustering analysis, and correlation analysis between immunocyte and 5mC regulators in each cluster was conducted.

**Results:**

Nine hub 5mC regulators were identified. A robust model was constructed, and its prominent classification accuracy was verified *via* ROC curve analysis (area under the curve [AUC] = 0.936 in the training cohort and AUC = 0.888 in the external validation cohort). Besides, the clinical effect of the model was validated by decision curve analysis. Then, 5mC modification clusters in AMI patients were identified, along with the immunocyte infiltration levels of each cluster. The correlation analysis found the strongest correlations were TET3—Mast cell in cluster-1 and TET3-MDSC in cluster-2.

**Conclusion:**

Nine hub 5mC regulators (*DNMT3B*, *MBD3*, *UHRF1*, *UHRF2*, *NTHL1*, *SMUG1*, *ZBTB33*, *TET1*, and *TET3*) formed a diagnostic model, and concomitant results unraveled the critical impact of 5mC regulators, providing interesting epigenetics findings in AMI population vs. stable CAD.

## 1 Introduction

Acute myocardial infarction (AMI) continues to be a common cardiac emergency incidence with significant morbidity and mortality worldwide and is caused by the rupture or erosion of a flawed, lipid-laden, chronic atherosclerotic coronary plaque, which causes an acute interruption of myocardial blood flow and ischemic myocardial necrosis (1, 2). According to prior findings, older individuals with coronary artery disease (CAD) have worse results, such as increased all-cause mortality and recurrent events (3, 4). Early and correct diagnosis may decrease mortality (5). Previous research reported some risk factors linked to the occurrence of AMI, including age, gender, alcohol use, diabetes, hypertension, physical labor, and smoking (6–9). However, there is growing evidence that genetics and epigenetics contribute to the occurrence and development of AMI (10).

Epigenetics is a collective term referring to processes that change the activity of the genome in a heritable way without affecting the DNA sequence (11). And the critical modification of 5-methylcytosine (5mC) is a dynamic and reversible modification process in epigenetics (12). DNA methyl transferase enzymes (writers) are responsible for DNA methylation in mammalian cells (13). They do so by adding a methyl group to the cytosine base’s carbon-5 position, which inhibits transcription in the genome (14). Researchers discovered that by altering DNA methylation, 5mC regulators (methyl transferase: writers; signal transducers: readers; and demethylase: erasers) are essential for a variety of cellular biological activities, including the silencing of retroelements, the stabilization of centrioles, and the regulation of gene expression (14–16). While it was reported that DNA methylation plays a major regulatory role in atherosclerosis, myocardial hypertrophy, heart failure as well as AMI (17–19), and diverse therapeutic targets for AMI and other diseases have been identified through the study of genetic factors (20–22). Studies examined 5mC regulators and molecular typing based on 5mC regulator gene expression are still limited. Consequently, research into new, highly sensitive and specific biomarkers for the diagnosis of cardiovascular disease is essential, and DNA methylation seems to be a promising new approach.

In this study, we systematically evaluate the modification pattern of 5mC regulators in AMI and CAD. Then, LASSO and SVM-RFE algorithms were employed to screen out the hub 5mC regulators. We also established a 5mC regulator-based classifier that can discriminate AMI from CAD *via* a machine-learning method. Afterward, we clustered AMI samples according to 21 5mC regulators, and two distinct 5mC modification clusters were identified. The immune cell differences as well as the correlations between hub 5mC regulators and 28 immunocytes were observed between the clusters. Besides, 64 5mC phenotype-related genes were identified and their biological functions were investigated. These findings may provide novel diagnostic biomarkers and a new perspective for individualized therapy in AMI patients.

## 2 Materials and methods

### 2.1 Ethics statement

This study gained the approval of the Ethics Committee of Guangdong Provincial People’s Hospital (GDREC2016255H). in accordance with the ethical standards of the Declaration of Helsinki. Written informed consent was collected from all participants.

### 2.2 Patient sample collections

We recruited 43 participants with complete information on biochemical and clinical parameters, and medical history, from Guangdong Provincial People’s Hospital between January 2022 and June 2022. The coronary angiography was performed on all patients, and two observers independently verified the angiographic results. Twenty-four patients diagnosed with AMI (23) were included in the test group, and nineteen patients diagnosed with Stable CAD (24) were included in the control group. The inclusion and exclusion criteria (25) and TIMI scores of the participants used in this research were in Supplementary Table 1. Blood samples were collected from each patient within 1 h from the admission, which were centrifuged at 2,000 rpm at 4°C for 30 min. The serum samples were isolated and stored at −80°C until analysis.

### 2.3 Animals

All animal experiments were conducted in compliance with the Guide for the Care and Use of Laboratory Animals by the US National Institutes of Health (NIH Publication No. 85-23, revised 1996) and approved by the Ethics Committee of Guangdong Provincial People’s Hospital. Sixty ApoE^–/–^ mice were housed in a pathogen-free environment at animal laboratory of Sun Yat-sen University. The animals were allowed access to food and water *ad libitum* on a 12-h light/dark cycle. ApoE^–/–^ mice were initially fed a standard rodent chow diet until 8 weeks of age and then switched to a high-fat diet (D12109C Formula) (New Brunswick, NJ). Afterward, the sixty mice were randomly divided into group A (AMI group, *n* = 30) and group C (CAD group, *n* = 30), They were anesthetized by intraperitoneal injection of sodium pentobarbital (50 mg/kg). AMI was performed on group A by ligation of the proximal left anterior descending coronary artery. Subsequently, the mice were sacrificed, and the infarcted myocardium in group A and the controls in group C were obtained for further experiments.

### 2.4 Western blots

The AMI and CAD tissues were lysed using strong RIPA buffer containing Halt Protease Inhibitor Cocktails (Thermo Fisher Scientific, Waltham, USA). Protein concentrations were evaluated with a bicinchoninic acid assay kit (Beyotime, Nantong, China). Primary antibodies targeting to beta actin (ab8226, Abcam), UHRF2 (ABE1028, MilliporeSigma), TET3 (ab153724, Abcam), UHRF2 (ZBTB33, MilliporeSigma), TET1 (ab19198, Abcam), DNMT3B (ab2851, Abcam), NTHL1 (ab191413, Abcam), UHRF1 (ab213223, Abcam), MBD3 (ab157464, Abcam), and SMUG1 (ab192240, Abcam), were incubated with targeted proteins at 4°C overnight, followed by incubating with appropriate horseradish peroxidase-conjugated secondary antibodies. Detection of horseradish peroxidase was performed with the Super Signal West Pico Chemiluminescent Substrate (Pierce).

### 2.5 RNA isolation and quantitative real-time PCR

Followed by total RNA extraction using TRIzol LS (Invitrogen) and examination of RNA quality and concentration using a NanoDrop ND-1000 analyzer according to the manufacturer’s instructions. Total RNA was subjected to reverse transcription using the GoScript™Reverse Transcription Mix (Promega). GAPDH was selected as an internal control gene, the primers are listed in Supplementary Table 2. Quantitative real-time PCR (qRT-PCR) was performed on Applied Biosystems QuantStudio 6 machine with SYBR-Green dye (Takara). The internal control was GAPDH, and data were calculated by the 2 ^(–ΔΔCt)^ method.

### 2.6 Public data obtaining

**FIGURE 1:**
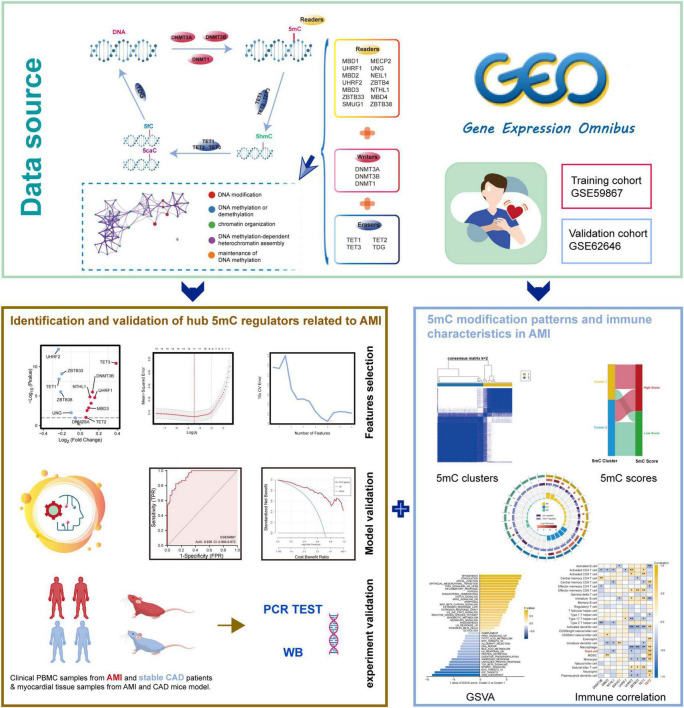
The illustrations for this study. The overall protocol utilized in the current study to identify hub 5mC regulators that can be potential biomarkers in unstable plaques and CAD progression and explore the characteristics of 5mC modification patterns in AMI.

The integration of bioinformatic analyses and experiment data is illustrated by the flowchart in Figure 1. Expression microarray data were downloaded from the Gene Expression Omnibus (GEO) database: GSE59867 dataset (26), which contained peripheral blood mononuclear cell (PBMC) samples from 111 patients with STEMI and 46 patients with stable CAD at admission; GSE62646 (27), which contained PBMC samples from 28 patients with STEMI and 14 patients with stable CAD at admission. The two independent datasets were both based on the GPL6244 platform of [HuGene-1_0-st] Affymetrix Human Gene 1.0 ST Array [transcript (gene) version]. Since AMI can hasten atherosclerosis 1 day after AMI, causing recurrent occurrence due to status altering of coronary artery walls or plaques (28), only admission patient data with STEMI and stable CAD were included in this study. We used GSE59867 (included 157 PBMC specimens at admission) in the screening of feature genes and as the training cohort during model construction, whereas GSE62646 was deployed as the external validation cohort (included 42 PBMC specimens at admission). In this article, 21 5mC regulators from previous studies (29–32) were systematically included: three writers (*DNMT1*, *DNMT3B*, and *DNMT3A*), 14 readers (*NEIL1*, *NTHL1*, *SMUG1*, *UHRF1*, *UHRF2*, *MBD1*, *MBD2*, *MBD3*, *MBD4*, *UNG*, and *MECP2*) and four erasers (*TDG*, *TET1*, *TET2*, and *TET3*).

### 2.7 Alteration analysis of 5mC regulator between AMI and CAD

The protein-protein interaction network of 21 5mC regulators was obtained from the STRING (33) database,^1^ and the PPI network was then visualized *via* Cytoscape (34) software, V3.8.3. Besides, the biological processes these 5mC regulators participate were analyzed by Metascape (35). Then, the expression value was pre-processed by the “normalize between arrays” function in the “limma” package (36), and differential gene expression analysis was conducted. A | log2FoldChange| value > 0, and a *p*-value < 0.05 were considered statistically significant. The expression relationship among 21 5mC regulators was evaluated by Spearman correlation analysis in AMI and CAD samples, respectively. Afterward, the expression status differences of 21 5mC regulators between AMI and CAD were compared by the Wilcox test.

### 2.8 Identification of hub AMI-related 5mC regulators and establishment of a classifier

The differentially expressed 5mC regulators were defined as the AMI-related 5mC regulators. Subsequently, these AMI-related 5mC regulators were subjected to least absolute shrinkage and selection operator (LASSO) regression (37) and support vector machine recursive feature elimination (38) (SVM-RFE) with 10-fold cross-validation, the R package “glmnet” and “e1071” helped implemented the above process. These two machine learning methods were used for feature selection and dimension reduction, so the hub AMI-related 5mC regulators were identified. Furthermore, by using “rms” package, we developed a 5mC regulator-related AMI-CAD classifier based on the hub 5mC regulator gene expression values, and receiver operating characteristic (ROC) curve analysis was used to evaluate the distinguishing performance of the classifier (39). Besides, a nomogram, a calibration plot, decision curve analysis (DCA) and a clinical impact curve were visualized based on the results, the above analyses can help assess the predictive power of the classifier and evaluated the clinical value of the classifier (40, 41).

### 2.9 Identification of 5mC-associated molecular clusters

To confirm distinct 5mC methylation modification patterns, unsupervised clustering analysis based on 21 5mC regulators expression profiles was conducted by the R package “ConsensuClusterPlus” (42). To guarantee the stability of the clustering, a thousand repetitions were performed, and each iteration contained 80% of the samples. The cumulative distribution function (CDF) curve of the consensus score was used to define the optimal cluster number. Then, similar to previous studies (43–47), principal component analysis was further performed on these 5mC regulators to calculate principal component 1, which was used for 5mC score calculation in this study.


5⁢m⁢C⁢s⁢c⁢o⁢r⁢e=Σ⁢P⁢C⁢1⁢i

i shows the expression of 5mC regulator genes.

### 2.10 Exploration of immune characteristics and hallmark pathway activity in the clusters

Gene set variation analysis (GSVA) *via* the R package “GSVA” was performed to explore Hallmark pathways on biological differences between two clusters (48). Besides, the enrichment scores that represented the 28 immunocytes infiltration levels in each sample were evaluated using single-sample GSEA (ssGSEA) algorithm, and immunocyte scores of the samples in CAD group were calculated as well. The marker genes of the 28 immunocytes were acquired from a previous study (49). The Kruskal-Wallis test was used to analyze the infiltration levels of each immunocyte between two clusters. Then the correlation between hub 5mC regulators and immunocytes was determined by Spearman correlation analysis. Subsequently, DEGs between clusters were identified by the R package “limma.” Then, Gene ontology (GO) and Kyoto Encyclopedia of Genes and Genomes (KEGG) functional enrichment analyses were conducted on these 5mC phenotype-related DEGs to observe the functions or pathways in which the 5mC regulators may affect.

### 2.11 Association of identified 5mC regulators and AMI

To further investigate the follow-up relationship between 5mC regulators and AMI. Two datasets (GSE59867 and GSE62646) were employed. The GSE59867 dataset contains relevant gene expression pattern at four time points of AMI patients: admission, discharge, after 1 month, and after 6 months. The GSE62646 dataset contains relevant gene expression pattern at three time points of AMI patients: admission, discharge, and after 6 months. The expression status of the hub 5mC regulators were compared between admission and other time points using Wilcoxon rank sum test.

### 2.12 Statistical analysis

R software 4.1.1 was conducted in this study for statistical analyses and visualization. The R package “ggplot2” was used to make statistical plots, and a two-tailed *P*-value < 0.05 was considered statistical significance unless otherwise specified.

## 3 Results

### 3.1 An overview of 5mC regulator genes in AMI and CAD

**FIGURE 2:**
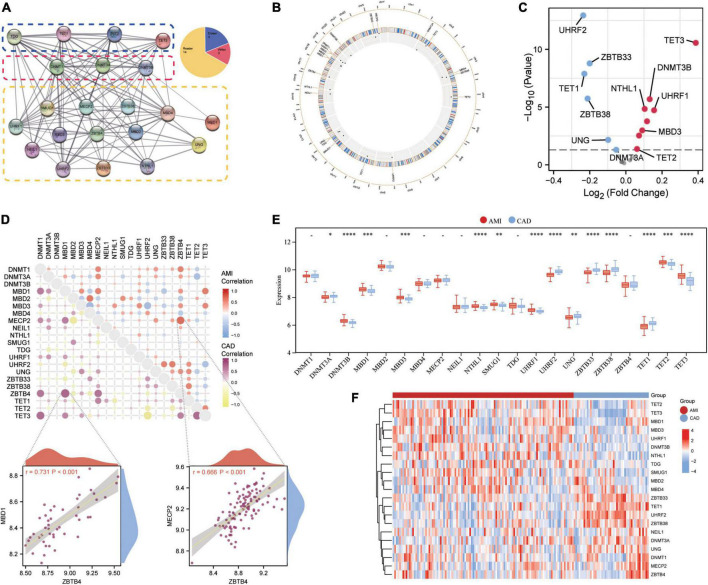
Expression landscape of 5mC regulators in AMI and CAD. **(A)** The protein-protein interactions among 21 5mC DNA methylation regulators and the composition summary of them. **(B)** Circle diagram of 5mC-related genes. **(C)** The volcano plot shows the expression changes of 21 5mC-related genes between AMI and CAD samples, and the statistically significant 14 5mC regulators are labeled. **(D)** Correlations among the expression of 21 5mC regulators in AMI and CAD samples, the correlation scatter plot shows the pair of genes with the most positive correlations in AMI and CAD, respectively. The box plot **(E)** and heatmap plot **(F)** demonstrated the transcriptome expression status of 21 5mC regulators between AMI and CAD samples.

Twenty-one 5mC regulators were investigated in this study, including 3 writers, 14 readers, and 4 erasers. A protein-protein network (Figure 2A) depicting the regulation interactions of these 5mC regulators was found to have multiple close connections. Besides, the biological processes these 5mC regulators take part in were exhibited in Supplementary Figure 1, and they are mainly involved in the DNA modification process just as anticipated. Figure 2B displays the location of the 5mC regulator genes *via* a circle diagram, which was mainly distributed on Chromosomes 2, 3, 12, 18, 19, and X. Besides, we analyzed the transcriptome links and discovered strong correlations between writers, readers, and erasers (Figure 2C): most regulators statistically correlated with each other in expression in both group of samples; in the CAD samples, reader MBD1 and reader ZBTB4 are the most correlated 5mC regulators; while in the AMI samples, reader MECP2 and read ZBTB4 showed the closest correlation, indicating these readers may function as a complex. Then, differentially expressed analysis found 14 dysregulated 5mC regulators, where eraser TET3 had the largest fold change, and the well-studied writer DNMT3A did not alter noticeably (Figure 2D), indicating that it might not be crucial in unstable plaques and CAD progression. The expression variations of the 21 5mC regulators were depicted in the boxplot (analyzed by the Wilcox test) and heatmap (Figures 2E, F), and the immunocytes alteration were shown in Supplementary Figure 2.

### 3.2 Hub 5mC regulator genes were selected *via* integrated machine learning methods

**FIGURE 3:**
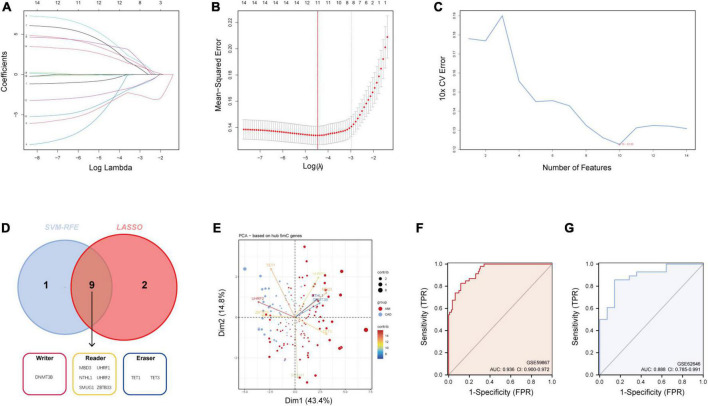
Identification of hub 5mC regulators. **(A)** The least absolute shrinkage and selection operator (LASSO) coefficient profiles of the 14 5mC regulators. **(B)** 10-fold cross-validation for optimum tuning parameter (λ) selection using LASSO. **(C)** Estimating 10-fold cross-validation error using the support vector machine recursive feature elimination (SVM-RFE). **(D)** Intersections of diagnostic gene outputs generated by LASSO and SVM-RFE. **(E)** Principal component analysis of nine hub 5mC regulators between AMI and CAD. The ROC curve and evaluated by the AUC value in the training cohort **(F)** and the external validation cohort **(G)** based on the constructed logistic model.

As mentioned earlier, 14 5mC regulators were dysregulated between AMI and CAD. Then, the abovementioned genes in the training cohort were used as inputs for both LASSO (Figures 3A, B) and the SVM-RFE algorithm (Figure 3C) with 10-fold cross-validation. By taking the intersection of the outputs of LASSO and SVM-RFE algorithms, nine hub 5mC regulator genes were identified (Figure 3D), including one writer (DNMT3B), six readers (MBD3, UHRF1, UHRF2, NTHL1, SMUG1, and ZBTB33), and two erasers (TET1 and TET3). Furthermore, a diverse 5mC regulator expression pattern between AMI and CAD was also shown by PCA results (Figure 3E), and the contribution of each hub regulator was shown: the erasers contribute more than other regulators, suggesting some potential roles of which in unstable plaques and CAD progression.

### 3.3 Construction and assessment of a logistic regression model for AMI diagnosis

**FIGURE 4:**
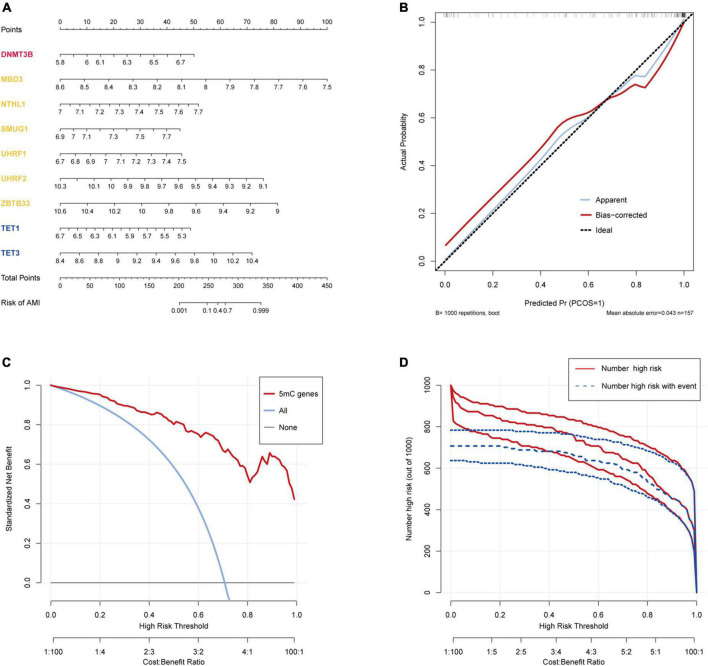
Construction and validation of a logistic model for AMI diagnosis based on the training dataset GSE59867. **(A)** Nomogram to predict the occurrence of AMI. **(B)** Calibration curve to assess the predictive power of the logistic model. **(C)** DCA curve to evaluate the clinical value of the logistic model. **(D)** Clinical impact curve based on the DCA curve to assess the logistic model.

**FIGURE 5:**
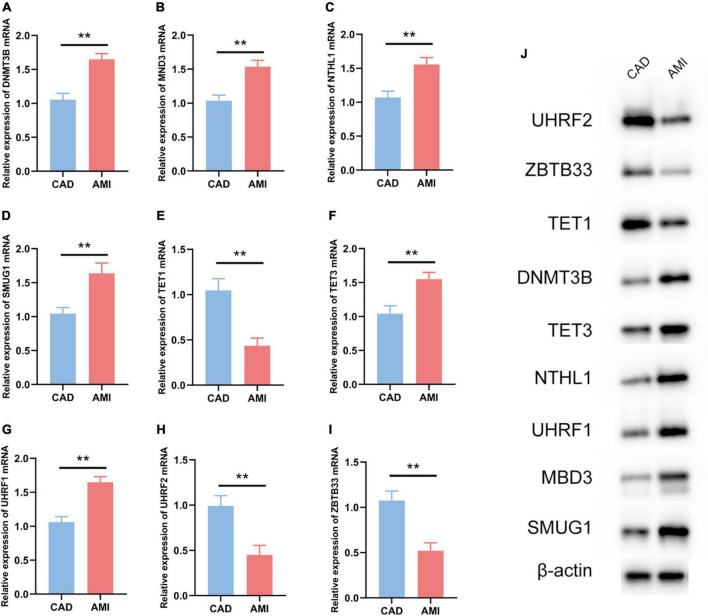
Experiment validation of the nine hub 5mC regulators. **(A–I)** DNMT3B, MND3, NTHL1, SMUG1, TET1, TET3, UHRF1, UHRF2, ZBTB33. The results were presented as mean ± standard deviation (*t*-testing). **p < 0.01, **(J)** Western blot analysis of hub 5mC regulators.

The R package “Rms” was utilized to constructed a logistic regression model for AMI diagnosis based on the hub 5mC regulators (DNMT3B, MBD3, UHRF1, UHRF2, NTHL1, SMUG1, ZBTB33, TET1, and TET3). The ROC curve was plotted (Figures 3F, G), and the classification model showed a satisfactory discrimination capability in both the training cohort (area under the curve [AUC] = 0.936, concordance index [CI] = 0.900–0.972) and the external validation cohort (AUC = 0.888, CI = 0.785–0.991). According to the logistic regression model, a nomogram was generated (Figure 4A). Afterward, a calibration curve was plotted to assess the predictive capability of the classifier and very little difference between the actual and predicted AMI risks was shown, demonstrating the robustness of the model (Figure 4B). Besides, the DCA of the model was conducted, and patients who use this model would be more beneficial than either the treat-none or the treat-all scheme (Figure 4C). Furthermore, the clinical impact curve based on the DCA curve was plotted to test the clinical influence of the logistic regression model (Figure 4D). At high risk threshold from 0.4 to 1, the “Number high risk with event” curve was close to the “Number high risk” curve, suggesting that the logistic regression model has the exceptional predictive capability. In certain ways, these findings also suggested that the 10 hub 5mC regulators may be crucial in unstable plaques and CAD progression. To validate our findings, experiments were conducted, and qRT-PCR (Figures 5A–I) and Western blot (WB) (Figure 5J) results showed significantly differentiated expressions of the genes between AMI and CAD clinical samples, which were consistent with our bioinformatics results.

In addition, information on the association between genes change level and AMI outcome was also analyzed. Based on patients TIMI risk score, 5mC regulators’ expression level in patients with high, intermediate, low-risk score was evaluated (Supplementary Figure 3).

### 3.4 The association of identified nine 5mC regulators and AMI

**FIGURE 6:**
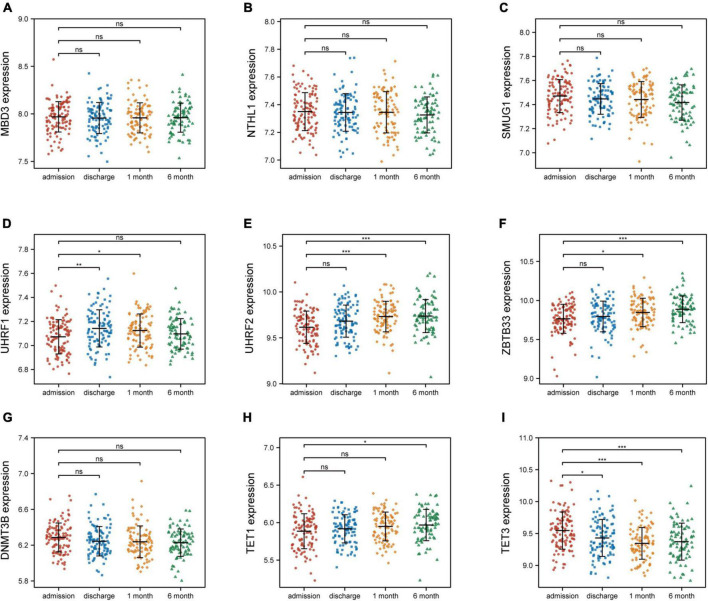
Nine 5mC regulators expression level at four time points of AMI patients in GSE59867. **(A–I)** MND3, NTHL1, SMUG1, UHRF1, UHRF2, ZBTB33, DNMT3B, TET1, TET3. *p < 0.05; **p < 0.01; ***p < 0.001; ns, not significant.

To investigate the relationship between these nine 5mC regulators and AMI, the expression status of these 5mC regulator genes were evaluated. Of interest, we found that the expression levels of “eraser” TET3 was the most significantly varied in both training cohort (Figure 6) and external validation cohort (Supplementary Figure 4).

### 3.5 5mC methylation modification patterns mediated by 21 regulators in AMI

**FIGURE 7:**
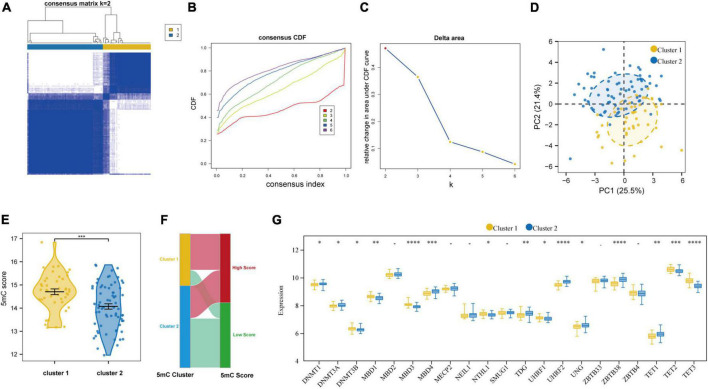
Identifying 2 distinct 5mC modification pattern clusters in AMI by unsupervised clustering of 21 5mC regulators. **(A)** Heatmap of the matrix of co-occurrence proportions for AMI specimens. **(B)** Consensus clustering cumulative distribution function (CDF) for *K* = 2–6. **(C)** Relative change in area under CDF curve for *K* = 2–6. **(D)** Principal component analysis for the transcriptome profiles of two 5mC clusters. **(E)** The differences in the 5mC score between the 5mC clusters. **(F)** Sankey plot showing the correlations between the 5mC score groups and 5mC clusters. **(G)** The expression status of 21 5mC regulators between the two 5mC clusters. *p < 0.05; ^**^p < 0.01; ^***^p < 0.001; ^*⁣*⁣**^p < 0.0001.

Based on the expression levels of 21 5mC regulators in AMI, the pam clustering algorithm with two clusters (including 43 samples in cluster-1 and 68 samples in cluster-2) was found achieved the clearest population clusters, and *k* = 2 was determined as the optimal value (Figures 7A–C), and the detail of the clusters and samples were listed in Supplementary Table 2. Besides, the PCA results showed distinct 5mC modification patterns between the two clusters (Figure 7D), and the 5mC score of each sample was calculated based on the abovementioned calculation formula. We found that cluster-1 members have a much higher 5mC score than cluster-2 members (Figure 7E), and the correlations between the 5mC score samples and 5mC cluster samples were shown by a Sankey diagram (Figure 7F). Afterward, the expression values of the 21 5mC regulators were compared, and the majority of them notably altered between cluster-1 and cluster-2, demonstrating distinct expression patterns between the two clusters (Figure 7G).

### 3.6 Function and immunocyte infiltration analysis based on molecular typing

**FIGURE 8:**
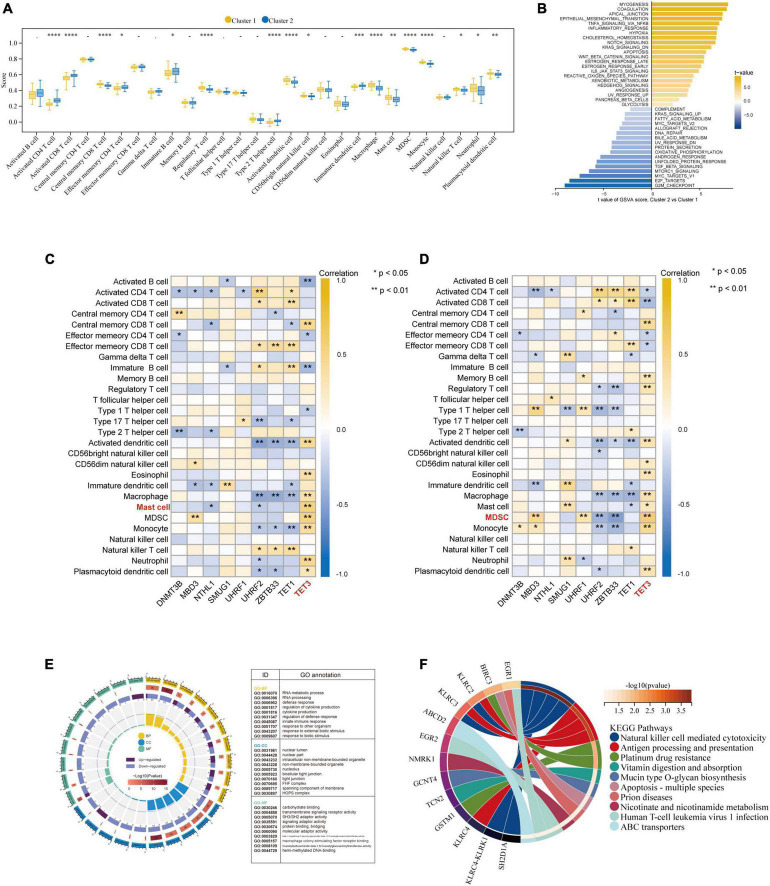
Further exploration of the characteristics between the two AMI clusters. **(A)** The abundance differences of each infiltrating immunocyte in 2 5mC modification clusters. **(B)** The differences in HALLMARKS pathway enrichment score between 5mC modification cluster-1 and cluster-2. The correlation between 28 immunocytes and hub 5mC regulators in cluster-1 **(C)**, and cluster-2 **(D)**. **(E)** Gene Ontology enrichment analysis of DEGs between cluster-1 and cluster-2, the outermost ring represented the name of pathways. The second outer ring represented the number of genes in pathways, and the heights of the columns in the inner ring indicate the value of GeneRatio. **(F)** Chord plot depicting the relationship between genes and KEGG signaling pathways. *p < 0.05; ^**^p < 0.01.

Besides, based on the ssGSEA results, the enriched scores of 28 types of immunocytes were evaluated, and some immunocytes differ between the two subtypes. For example, cluster-1 owned a greater monocyte count, while cluster-2 owned a greater immature and activated B cell count (Figure 8A). The distinct immunological features were explored, we then explored other biological functions between the two clusters. The different HALLMARK pathways activity between the clusters were analyzed *via* GSVA algorithm (Figure 8B), and we found that the MYOGENESIS pathway and the G2M_CHECKPOINT pathway were the most significantly dysregulated ones, indicating their potential linkages. Then, the correlation between immunocytes and hub 5mC regulators were analyzed in separate cluster (Figures 8C, D), the results show that the most positively correlated immunocyte and 5mC regulator pair in cluster-1 was TET3 and Mast cell whereas the most positively correlated immunocyte and 5mC regulator pair in cluster-2 was TET3 and Myeloid-derived suppressor cell (MDSC). Then 64 DEGs between the clusters were identified (Supplementary Figure 5), and these DEGs were subjected to GO and KEGG functional enrichment analyses (Figures 8E, F). The enrichment results showed that they were mainly involved in cytokine-related pathways and immune-related pathways.

## 4 Discussion

AMI, a leading cause of morbidity and mortality globally, is increasingly being researched by academics in terms of its early diagnosis and treatment (50). Prior studies reported the bidirectional relationship between plaques progression and CAD progression: stable CAD to STEMI can induce plaques progression and plaque progression can worsen CAD progression (28, 51). Nevertheless, the therapeutic targets and predictive biomarkers are currently few, and AMI continues to be a major factor in death and disability. Thus, more research is needed to find novel biomarkers for the early detection of AMI.

To add more theoretical bases to the urgent issue, here, we systematically investigated the 21 5mC regulators under the “stable CAD to AMI” circumstance. A set of bioinformatics algorithms were performed, and 14 differentially expressed 5mC regulators genes were identified which correlated and interacted with each other, resulting in a 5mC regulation network in AMI and CAD. Then, examined by the integrated machine learning methods (LASSO and SVM-RFE), the nine most important 5mC regulators (DNMT3B, MBD3, UHRF1, UHRF2, NTHL1, SMUG1, ZBTB33, TET1, and TET3) were screened out. Afterward, similar to previous studies (52, 53), a logistic model was established for the AMI diagnosis. Satisfactory discrimination ability was shown, for which C-index is 0.936 in the training cohort and 0.888 in the external validation cohort. The calibration, DCA and clinical impact curve plots indicated the pleasant fit of the model, suggesting DNA methylation may be a crucial molecular variable in the development of AMI.

Given the individual heterogeneity of 5mC methylation modification, unsupervised clustering of the AMI specimens based on 5mC regulator expression profiles was performed, and the results led us to two clusters with distinctive 5mC modification patterns, and distinct immunocyte characteristics were observed. Of interest, we found that many significant correlations between hub 5mC regulators and immune cells in the clusters, respectively. The most correlated pair in cluster-2 is TET3-MDSC (correlation coefficient = 0.65, *p* < 0.001), while TET3- Mast cell (correlation coefficient = 0.59, *p* < 0.001) in cluster-1. MDSCs are pathologically activated monocytes and neutrophils with formidable immunosuppressive activity (54), and current studies found that MDSCs mobilization were closely related to AMI (55). By preventing the local inflammatory response and inflammation-mediated apoptosis, MDSCs may have a positive and protective effect on the process of ventricular remodeling after AMI (56). However, Yao et al. reported that myocardial damage in AMI mice can get worse by the growing MDSCs infiltration (57). Besides, DNA methylation can in a way regulate MDSC metabolism. Smith et al. reported that MDSC survival could be affected by DNA methylation *via* an independent mechanism (58). According to our findings, a tight bond of mast cell and 5mC regulator was revealed in AMI subclusters. Mast cells have been seen in AMI and are multifunctional cells that contain a variety of mediators, including histamine, tryptase, and cytokines (59). Kupreishvili et al. reported that excessive mast cell infiltration can give rise to increased risk of AMI, possibly increasing the risk of re-infarction (60). In addition, Leoni et al. found that DNA methylation is crucial in regulating mast cell reactivity (61). Regarding TET3, i.e., tet methylcytosine dioxygenase 3, which is one of the DNA methylation eraser regulators and can oxidize 5mC into 5-hydroxymethylcytosine (5hmC) (62). It has been verified that TET3 was closely associated with the process of stem cell renewal, epigenetic modulation, tumor, and embryonic development (63–66). However, the relationship between TET3 and AMI or between TET3 and MDSC is yet to be found. Furthermore, based on the GSVA results, distinct pathway overview between clusters were drawn. As demonstrated by a previous literature, the pathophysiology of heart failure (HF) is caused by the inappropriate resolution of inflammation following post-myocardial injury, which is linked to failed left ventricular remodeling (67). Apparently, immune-related pathways were more activated in cluster-1, suggesting patients in this cluster may suffer a poor prognosis. Last, we identified the 5mC phenotype-related genes, and the GO and KEGG enrichment analyses results were mainly involved in cytokine-related pathways and immune-related pathways, indicating these processes may mainly contribute to 5mC mediation in AMI. Abundant discoveries were found, and other researchers in the field will get directions to catch the key 5mC regulator and immune features in AMI rapidly.

To our best knowledge, our study is the first one to systematically explored the biomarkers for stable CAD to AMI progression regarded to 5mC regulators. Fruitful findings were generated: hub 5mC regulators were identified and validated by qRT-PCR; an AMI diagnosis model was built and validated; subclusters of AMI were identified, as well as their unique immune characteristics.

### 4.1 Limitations of the present study

However, there are still some limitations that we must admit. This research is mainly based upon silico analysis, and most findings are theoretically sound but haven’t been tested in actual experiments. Although nine hub 5mC regulators were validated by a robust model, an external validation cohort, and qRT-PCR, the biological function and specific mechanism they may involve in AMI is still a giant gap. Besides, the method for immunocyte infiltration analysis is based on the most widely applied ssGSEA algorithm, although single-cell sequencing is still needed to obtain the most precise number of immunocytes. More importantly, some important information were not reported in the current study, such as biomarkers, echo, EKG and clinical characteristics of the population.

## 5 Conclusion

In brief, a non-negligible impact of 5mC regulators on the diagnostic effect of stable CAD to AMI was determined. Nine hub 5mC regulators are identified to be latent biomarkers in AMI (DNMT3B, MBD3, UHRF1, UHRF2, NTHL1, SMUG1, ZBTB33, TET1, and TET3). Besides, two 5mC molecular clusters were identified, and the immunocyte infiltration and pathway activity of each cluster was analyzed in this study. The findings may provide a novel direction for the follow-up exploration of the molecular mechanism of 5mC regulators in the progression of stable CAD to AMI and provide a new reference for the personalized treatment of patients.

## Data availability statement

Publicly available datasets were analyzed in this study. This data can be found here: https://www.ncbi.nlm.nih.gov/geo/query/acc.cgi.

## Ethics statement

This study gained the approval of the Ethics Committee of Guangdong Provincial People’s Hospital. The patients/participants provided their written informed consent to participate in this study.

## Author contributions

QH, XD, and YL: conceptualization and design. YG, HJ, JW, and PL: formal analysis, visualization, and manuscript writing. PL: sample collection. XZ: data processing and revision. YG and HJ: animal experiments. TZ, RN, and JF: writing – review and editing. All authors critically reviewed and approved the final version of the work.

## Footnotes

1
https://string-db.org/
