# Supplementary material for: 5mC modification patterns provide novel direction for early acute myocardial infarction detection and personalized therapy
Source: Front Cardiovasc Med. 2022 Dec 23;9:1053697. doi: 10.3389/fcvm.2022.1053697 (PMC9816341; doi:10.3389/fcvm.2022.1053697)
Supplement: Supplementary file 1 [file Table_1.DOCX]

# Supplementary materials

# Supplementary figures (figure S1-S5)

**Supplementary Figure 1**. GO-biological process enrichment analysis of the 21 5mC regulators. Terms with a similarity score > 0.3 are linked by an edge (the thickness of the edge represents the similarity score). The network is visualized with Cytoscape with “force-directed” layout and with edge bundled for clarity. One term from each cluster is selected to have its term description shown as label.
**Supplementary Figure 2**. The abundance differences of each infiltrating immunocyte in AMI and CAD patients.

**Supplementary Figure 3**. 5mC regulators’ expression level in patients with high, intermediate, low-risk TIMI score

**Supplementary Figure 4**. Nine 5mC regulators expression level at three time points of AMI patients in GSE62646. (A-I): MND3, NTHL1, SMUG1, UHRF1, UHRF2, ZBTB33, DNMT3B, TET1, TET3. *p < .05; **p < .01; ***p < .001; ns: not significant.

**Supplementary Figure 5**. volcano plot shows the differentially expressed genes (cluster-1 vs cluster-2).

# Supplementary tables (Table S1-S3)

**Supplementary Table 1**. The inclusion and exclusion criteria of clinical participants, and clinical characteristics of the participants.

**Supplementary Table 2**. Primer sequences for qRT-PCR.

**Supplementary Table 3**. The 5mc clusters of patients with AMI.
