# Supplementary figures and images for: 5mC modification patterns provide novel direction for early acute myocardial infarction detection and personalized therapy
Source: Front Cardiovasc Med. 2022 Dec 23;9:1053697. doi: 10.3389/fcvm.2022.1053697 (PMC9816341; doi:10.3389/fcvm.2022.1053697)

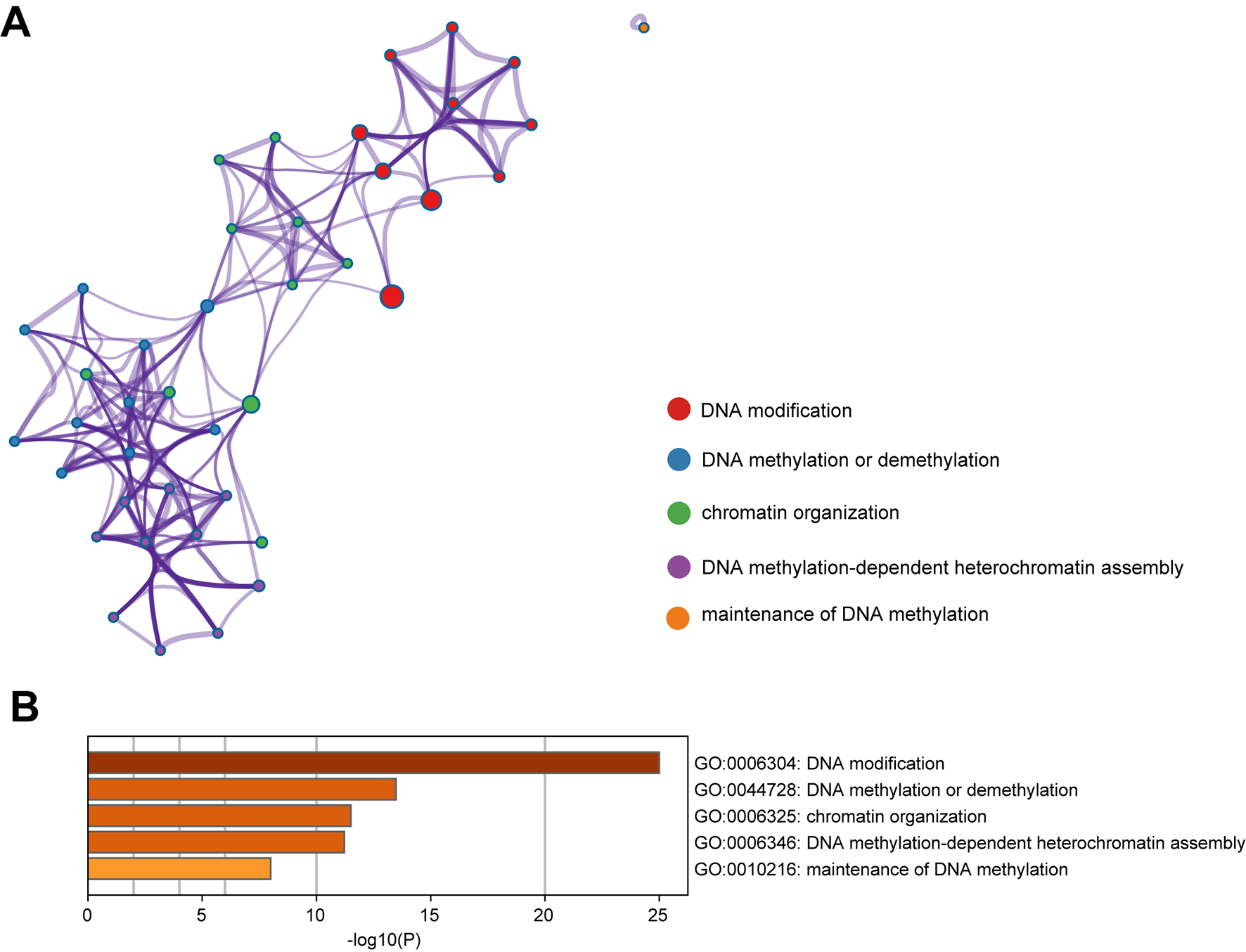

Supplement: Supplementary file 3 [file Image_1.TIF]

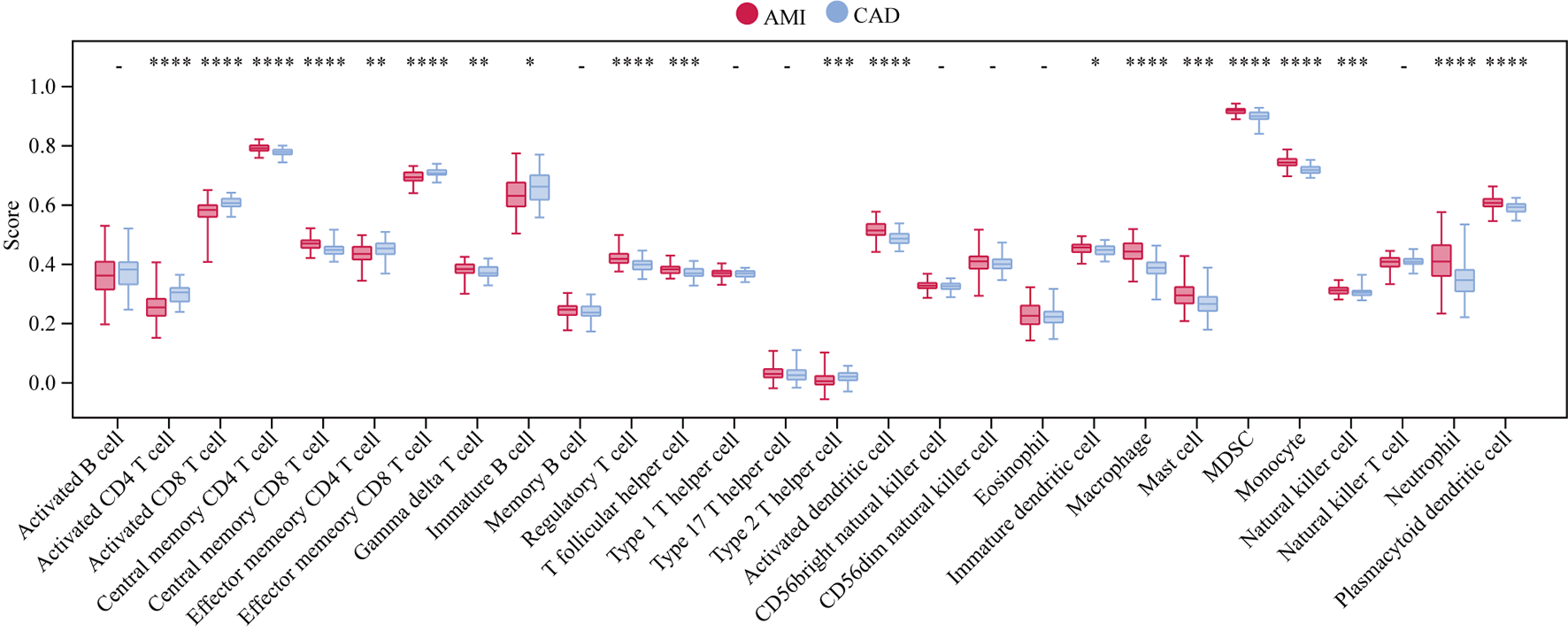

Supplement: Supplementary file 4 [file Image_2.TIF]

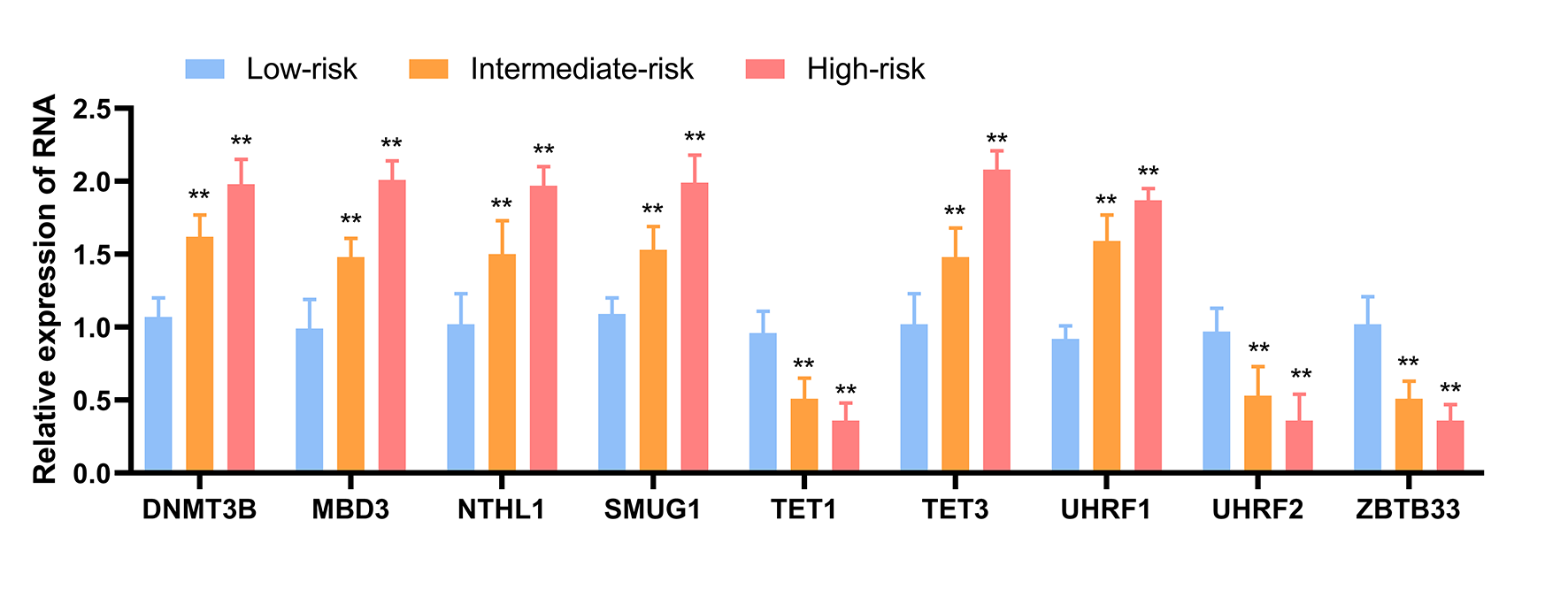

Supplement: Supplementary file 5 [file Image_3.TIF]

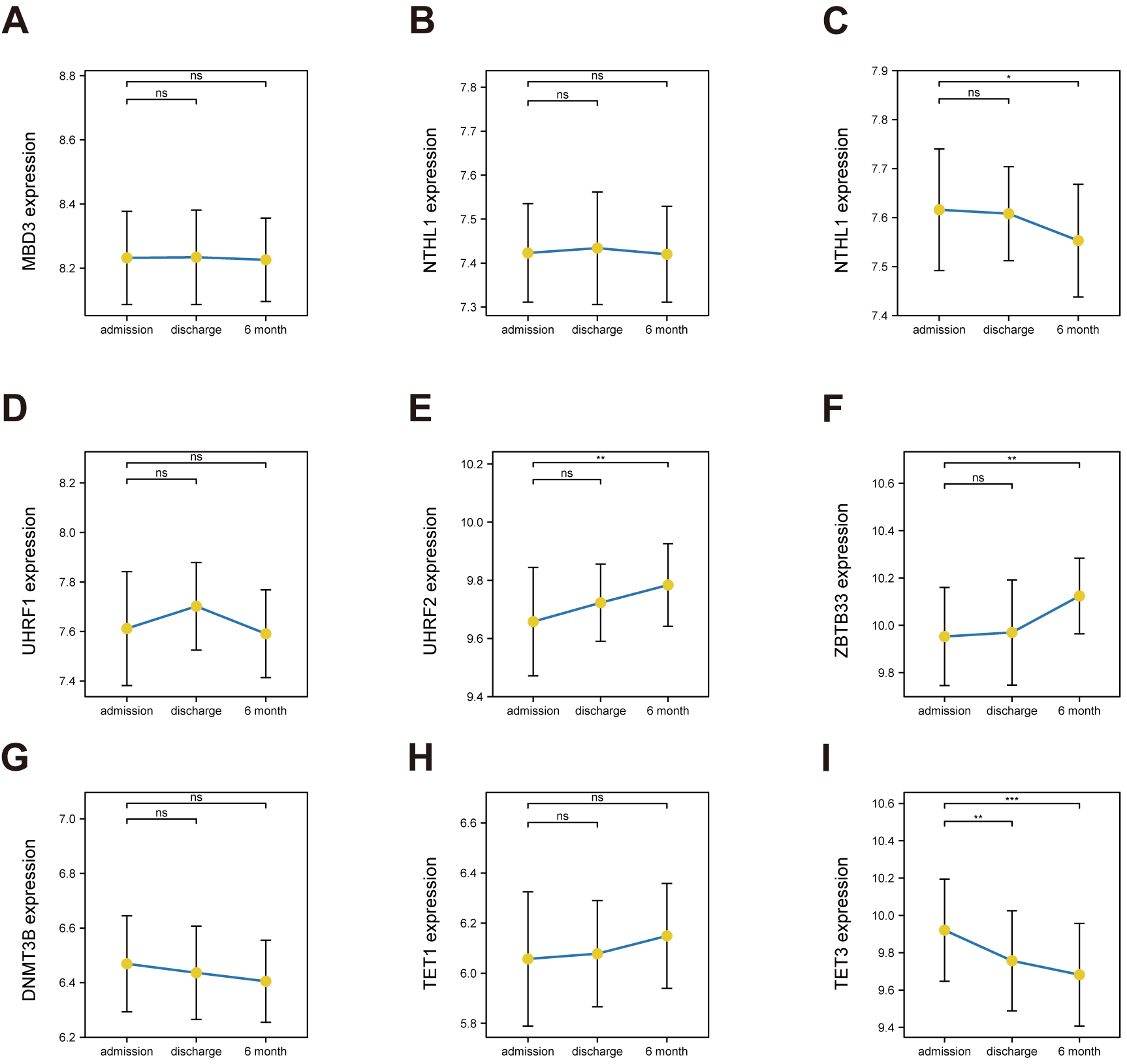

Supplement: Supplementary file 6 [file Image_4.TIF]

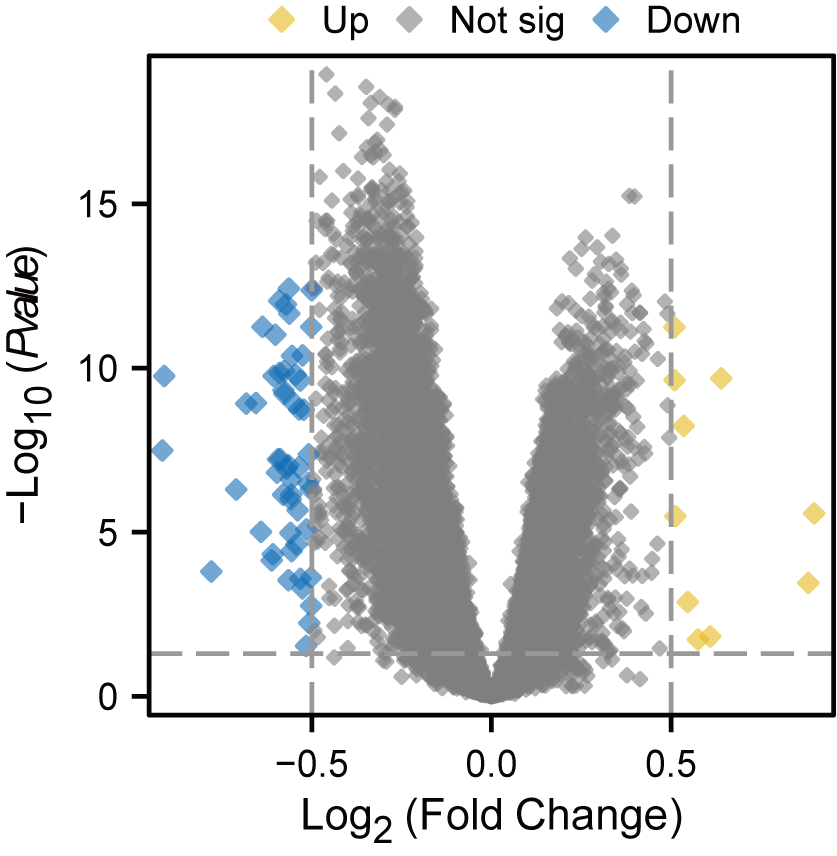

Supplement: Supplementary file 7 [file Image_5.TIF]
